# Single-shot third-harmonic spectral diagnostics for tender X-ray FEL pulses using a bent-crystal spectrometer

**DOI:** 10.1107/S1600577526006739

**Published:** 2026-08-28

**Authors:** Christopher Arrell, Henrik Lemke, Eduard Prat

**Affiliations:** a Center for Photon Science Paul Scherrer Institut CH-5232Villigen PSI Switzerland; b Center for Accelerator Science and Engineering Paul Scherrer Institut CH-5232Villigen PSI Switzerland; European XFEL, Germany

**Keywords:** tender X-rays, FELs, spectrometers

## Abstract

The tender X-ray (2.0–4.0 keV) region provides access to the *K*-edges of light- and mid-*Z* elements that are central to catalysis, energy materials and soft condensed matter, but direct single-shot spectral diagnostics in this range are technically challenging. Ruled gratings typically offer limited resolving power, while scattering-based approaches suffer from low efficiency in an on-line geometry. We demonstrate that a bent-crystal spectrometer on the SwissFEL Aramis beamline can be adapted to provide indirect single-shot spectral diagnostics for tender X-ray operation by measuring the natural third harmonic of 2.1 and 2.5 keV self-amplified spontaneous emission pulses at 6.3 and 7.5 keV, respectively. The scaled third-harmonic spectra reproduce the bandwidth obtained from independent monochromator scans of the fundamental, yielding a relative bandwidth of Δ*E*/*E* ≃ 3.1‰ at 2.5 keV. An autocorrelation analysis of 2400 shots gives an upper limit on the instrumental resolution better than 6 × 10^−5^ at 7.5 keV. The two-dimensional spectral images further reveal the transverse-mode structure and provide sensitive real-time feedback for optimizing free-electron laser performance in the tender X-ray regime.

## Abstract 2

Third-harmonic spectral monitoring with an existing bent-crystal spectrometer provides single-shot bandwidth diagnostics and real-time machine tuning for tender X-ray free-electron laser operation, and can be made non-invasive when combined with a suitable beam-sampling grating.

## 1. Introduction

At X-ray free-electron laser (XFEL) facilities, operational energy ranges increasingly extend into the tender X-ray region (2.0–4.0 keV) (Prat *et al.*, 2020 ▸; Park *et al.*, 2025 ▸). This window contains the *K*-edges of key light- and mid-*Z* elements such as P (2.15 keV), S (2.47 keV), Cl (2.82 keV) and Ca (4.04 keV), as well as *L*-edges of 4*d* transition metals such as Ru (2.84 keV), Rh (3.00 keV) and Pd (3.17 keV), which are central to catalysis, organic electronics and biological systems. Access to these edges enables element-specific X-ray spectroscopies (XANES/EXAFS) and resonant scattering or diffraction experiments with enhanced contrast and chemical sensitivity (Freychet *et al.*, 2021 ▸; McNeill, 2023 ▸). Applications already span battery and photovoltaic materials, metalloproteins, and homogeneous/heterogeneous catalysis, where time-resolved tender X-ray absorption and scattering have been used to follow metal–ligand bonding and C–H activation dynamics in solution and at interfaces (Jay *et al.*, 2023 ▸; Cutsail III & DeBeer, 2022 ▸).

Realizing this science at FELs requires precise control and on-line monitoring of the X-ray pulse properties. In particular, single-shot measurements of the spectral distribution are essential for normalizing data and optimizing self-amplified spontaneous emission (SASE) operation. For hard X-rays, single-shot spectral diagnostics have been implemented in two main configurations. In the first, a thin bent crystal is placed directly in the FEL beam and disperses the small fraction of radiation it diffracts onto a position-sensitive detector while transmitting the bulk of the pulse to downstream experiments (Zhu *et al.*, 2012 ▸; Boesenberg *et al.*, 2017 ▸). In the second, a thin transmission grating placed upstream of the bent crystal diffracts a small portion of the beam into a side branch where the bent crystal then provides the spectral dispersion, yielding a fully non-invasive on-line bent-crystal spectrometer; this architecture has been implemented at SACLA (Katayama *et al.*, 2016 ▸; Makita *et al.*, 2015 ▸), the European XFEL (Kujala *et al.*, 2020 ▸) and SwissFEL (Rehanek *et al.*, 2017 ▸; David *et al.*, 2021 ▸), covering photon energies from ∼4 to 13 keV (and to 25 keV at the European XFEL). In the tender X-ray regime, however, on-line single-shot diagnostics remain more challenging. Ruled gratings tend to offer reduced resolving power and efficiency in the 2–4 keV range, whereas dedicated scattering-based von Hamos spectrometers provide high resolution but require additional space and hardware and typically operate with lower signal-to-noise when used in an on-line geometry (Rehanek *et al.*, 2018 ▸). Moreover, the existing SwissFEL bent-crystal spectrometer geometry is incompatible with direct tender X-ray measurements, since the dispersed beam exits through a beryllium window and then propagates over an air or helium path of order 1 m, leading to strong attenuation below ∼4 keV.

An attractive route to overcome this gap is to exploit the natural third-harmonic emission produced during SASE operation. When the FEL is tuned to a fundamental photon energy in the tender X-ray region (*e.g.* ∼2.5 keV), the corresponding third harmonic lies in the hard X-ray range (∼7.5 keV), squarely within the acceptance and transmission window of bent-crystal-based single-shot spectrometers. If the third-harmonic spectrum faithfully reflects the shot-to-shot bandwidth and fluctuations of the fundamental, it can serve as a proxy diagnostic for tender X-ray operation using existing hard X-ray beamline instrumentation, and, when combined with a suitable beam-sampling grating, as a non-invasive on-line diagnostic.

In this communication, we demonstrate the adaptation of the SwissFEL beamline bent-crystal spectrometer to acquire high-fidelity single-shot spectra of the third-harmonic FEL emission during operation in the tender X-ray regime. Using a two-dimensional complementary metal oxide semiconductor (CMOS) detector, we access both the spectral bandwidth and the transverse spectral distribution of the third harmonic. We then confirm that the relative spectral bandwidth of the third harmonic matches that of the fundamental tender X-ray FEL emission by comparing the bent-crystal measurements with independent bandwidth determinations obtained from scans of the beamline monochromator over the fundamental. This establishes third-harmonic spectral monitoring with bent crystals as a practical on-line diagnostic tool for tender X-ray FEL pulses, without requiring additional in-vacuum optics or modifications to the primary beam path.

## 2. Theory

### 2.1. Fundamental and third-harmonic FEL bandwidth

To justify the use of the third harmonic as a proxy for the tender X-ray fundamental, we briefly recall the scaling of bandwidth and coherence in a SASE FEL. In a SASE XFEL operating near saturation, the fundamental exhibits a characteristic relative bandwidth close to the Pierce parameter ρ (Bonifacio *et al.*, 1984 ▸), which is typically between 10^−4^ and 10^−3^ for X-rays. Time-dependent simulations of nonlinear harmonic generation in planar undulators show that, at saturation, the relative spectral bandwidth Δ*E*/*E* is essentially the same for all odd harmonics of the SASE radiation (Saldin *et al.*, 2006 ▸). Because the harmonics are generated by the same microbunched electron beam, they inherit its longitudinal structure: theoretical studies find that the coherence time at saturation decreases approximately inversely with harmonic number, while the relative bandwidth remains nearly harmonic independent (Saldin *et al.*, 2006 ▸; Huang & Kim, 2007 ▸; Schneidmiller & Yurkov, 2012*a* ▸). In other words, although the third-harmonic photon energy is three times the fundamental, the third-harmonic spectrum at saturation has an absolute width Δ*E*_3_ ≃ 3 Δ*E*_1_, so that the scaled width Δ*E*_3_/(3*E*_1_) closely matches Δ*E*_1_/*E*_1_, and a single-shot measurement of the third-harmonic bandwidth, divided by the harmonic number, provides a direct estimate of the fundamental SASE bandwidth.

It should be noted that the scaling τ_coh, *h*_ ∝ 1/*h*, where τ_coh, *h*_ is the coherence time of the *h*th harmonic and *h* is the harmonic number, and the associated conclusion that the relative bandwidth is harmonic independent, are strictly valid in the exponential-gain regime approaching, but not yet reaching, saturation. At and beyond the saturation point, the simple proportionality *P*_*h*_ ∝ |*b*|^*h*^ breaks down as overbunching occurs at the peak of each temporal spike while the flanks continue to grow, analogous to the behaviour of a seeded harmonic-generation scheme driven slightly above the optimum seed power (Schneidmiller & Yurkov, 2016 ▸). This effect can narrow the coherence bandwidth of the harmonics relative to the pre-saturation prediction. In practice, however, a residual energy chirp on the electron bunch tends to restore a comparable relative bandwidth across the harmonics, and experimental measurements at operating FELs confirm that the scaled third-harmonic and fundamental bandwidths agree within the shot-to-shot fluctuations of the SASE process (Bidhendi *et al.*, 2025 ▸).

Experimental studies of nonlinear harmonic generation in SASE FELs support this picture: measurements at vacuum ultraviolet (VUV) and soft X-ray FELs have shown that the scaled third-harmonic spectra agree with the fundamental spectra in width within the shot-to-shot fluctuations (Bidhendi *et al.*, 2025 ▸). In the context of the current work, this implies that a hard X-ray spectrometer tuned to the third harmonic can serve as a reliable on-line bandwidth diagnostic for the tender X-ray fundamental, even when direct measurements of the fundamental are impractical.

### 2.2. Mode structure and coherence of fundamental versus harmonics

While the fundamental SASE radiation at saturation is close to diffraction-limited and dominated by the lowest-order transverse mode, the higher harmonics exhibit reduced coherence and a more complex mode structure. For typical XFEL parameters, three-dimensional simulations predict a degree of transverse coherence of the fundamental of ζ ≃ 0.8–0.9 at saturation, reflecting effective transverse-mode selection (Saldin *et al.*, 2003 ▸), and interferometric measurements at FLASH have confirmed values in this range (Roling *et al.*, 2011 ▸). The third harmonic, generated nonlinearly from higher Fourier components of the electron microbunching, does not undergo the same efficient filtering and is emitted into a broader angular distribution with a superposition of transverse modes.

Quantitatively, simulations for an XFEL in the hard X-ray regime show a degree of transverse coherence of about ζ_1_ ≃ 0.86 for the fundamental and ζ_3_ ≃ 0.72 for the third harmonic at the saturation point (Schneidmiller & Yurkov, 2016 ▸). Because the harmonic radiation power scales approximately as the *h*th power of the microbunching, the effective transverse source size of the third harmonic is smaller than that of the fundamental, but this narrower source radiates into a larger divergence, consistent with reduced transverse coherence (Saldin *et al.*, 2010 ▸; Schneidmiller & Yurkov, 2012*a* ▸). A second related effect is that the normalized emittance condition ɛ_*n*_/γ ≤ λ/4π that ensures diffraction-limited emission may be satisfied at the fundamental wavelength but not at the harmonic, since λ scales as 1/*h* while ɛ_*n*_ is a fixed property of the electron bunch (Schneidmiller & Yurkov, 2012*b* ▸; Saldin *et al.*, 1999 ▸). Once this condition is violated, the beam can no longer radiate efficiently into the fundamental transverse mode, further reducing the degree of transverse coherence at higher harmonics. Longitudinally, the coherence time of the odd harmonics at saturation scales approximately as 1/*h*, where *h* is the harmonic number, so that a third-harmonic pulse consists of shorter temporal spikes and exhibits a more pronounced multi-spike spectral structure (Saldin *et al.*, 2006 ▸; Schneidmiller & Yurkov, 2012*a* ▸; Huang & Kim, 2007 ▸).

From a photon-diagnostics perspective, these coherence properties have two practical consequences. First, the third-harmonic spectrum still provides an accurate measure of the relative bandwidth of the fundamental, as discussed above. Second, the reduced transverse coherence and multimode content of the third harmonic naturally lead to a grainier spatial pattern in single-shot images, with multiple beamlets and fine structure across the profile, rather than a single smooth mode. This behaviour is fully consistent with SASE theory and with previous analyses of the coherence of higher harmonics (Saldin *et al.*, 2003 ▸; Schneidmiller & Yurkov, 2012*a* ▸), and it underpins the interpretation of the structured third-harmonic angular distributions observed in our spectrometer.

## 3. Design

### 3.1. Adaptation to existing spectrometer

The existing bent-crystal spectrometer on the hard and tender X-ray FEL beamline (Aramis) at SwissFEL was originally designed to measure high-resolution single-shot spectra between 4 and 13 keV with a typical relative bandwidth of ∼1.5‰. For the measurements presented here, a cylindrically bent Si(220) crystal with a radius of curvature of 75 mm mounted on a motorized diffractometer was inserted into the X-ray beam, and the X-ray imaging system was positioned 1.5 m downstream along the dispersion direction. For operation at 7.5 keV (the third harmonic of a 2.5 keV fundamental), the corresponding Bragg angle of the Si(220) reflection is θ_B_ ≃ 25.5°. At this 1.5 m detector distance the spectrum is spread over a footprint that yields a signal per pixel too low for robust single-shot measurements of the third harmonic (see Section 3.2 for the calibrated dispersion).

**Figure 1:**
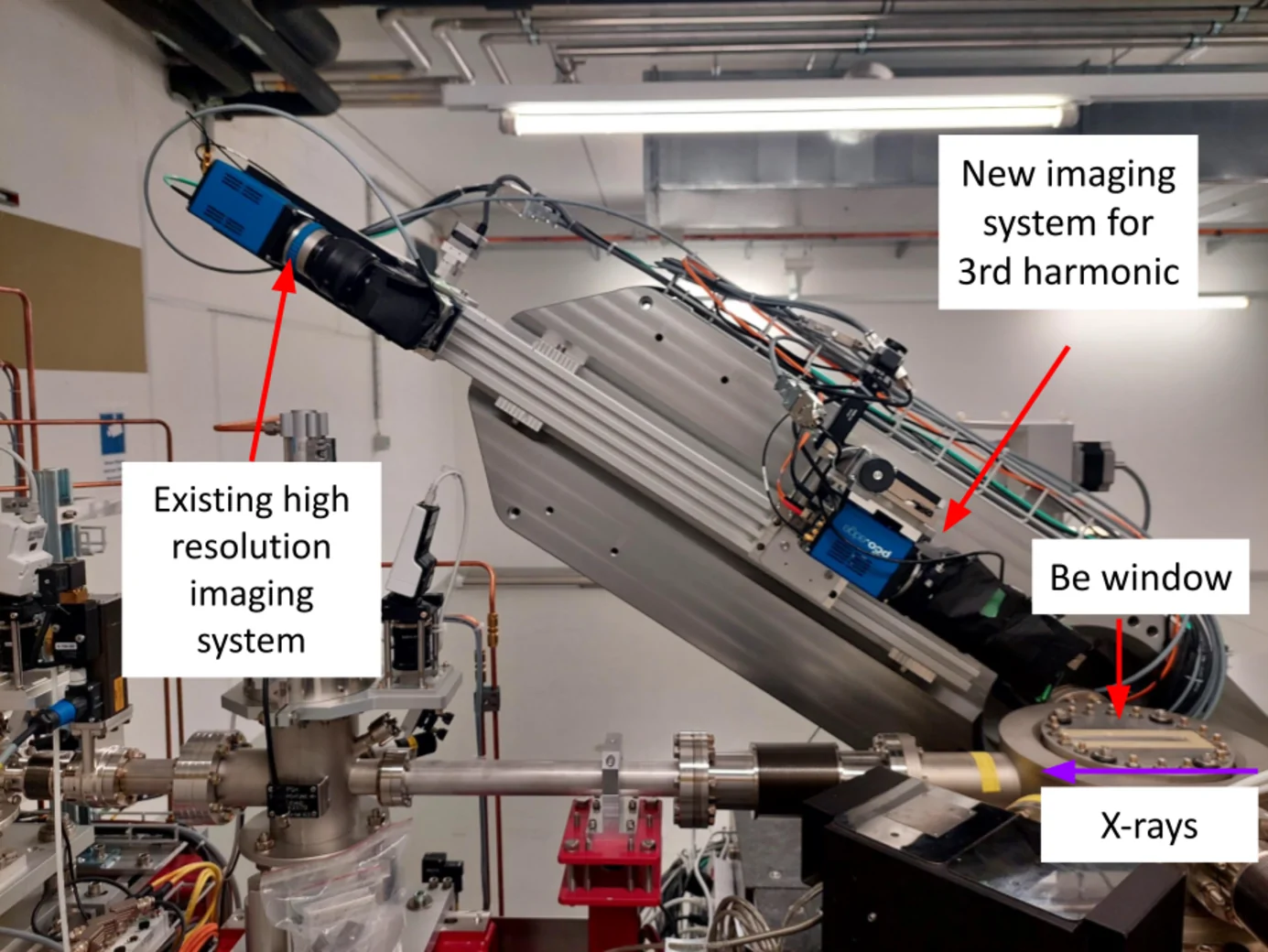
Side view of the additional imaging system mounted on the bent-crystal spectrometer diffractometer arm at SwissFEL. The insertable Ce:YAG/CMOS camera module is positioned at ∼0.25 m from the bent Si(220) crystal and can be translated in and out of the beam to record single-shot third-harmonic spectra without changing the original 1.5 m detector geometry. The original far-field detector setup at 1.5 m remains available for standard bent-crystal spectrometer operation in the 4–13 keV range.

To increase the photon flux density per pixel while keeping the same dispersive element and reflection, an additional insertable imaging system was mounted on the diffractometer arm at a distance of ∼0.25 m from the bent Si crystal (Fig. 1 ▸). At this shorter distance the energy dispersion remains sufficient to resolve the FEL bandwidth at 7.5 keV, while the spatial extent of the spectrum on the detector is reduced, increasing the signal per pixel by roughly an order of magnitude. The bent crystal is operated in the same geometry as in the standard bent-crystal spectrometer configuration, so that the energy is dispersed horizontally while maintaining approximate point-to-line focusing onto the detector plane.

Both imaging setups use a Ce:YAG scintillator to down-convert the X-ray photons to visible light. The Ce:YAG surface is imaged onto a pco.edge CMOS camera with a 5 mm × 5 mm field of view and an effective spatial sampling of 10 µm per pixel. The combined CMOS and Ce:YAG assembly is motorized both perpendicular to, and along, the diffractometer axis of rotation. This allows the closer imaging system to be inserted to record the dispersed X-rays at 0.25 m, and then retracted to recover the original 1.5 m detector configuration, without realigning the diffractometer for a given X-ray energy.

### 3.2. Spectrometer calibration and detector choice

The energy dispersion of the spectrometer was calibrated by scanning the SwissFEL undulator gap to vary the FEL central photon energy by ±20 eV around the nominal value in 5 eV steps and tracking the corresponding shift of the spectral peak on the detector. For the third harmonic at 7.5 keV with the Si(220) reflection at θ_B_ ≃ 25.5° and a crystal radius of curvature of 75 mm, the calibrated linear dispersion at the *L* = 0.25 m detector position is ∼64 meV per pixel of the CMOS sensor, corresponding to ∼29 meV µm^−1^ at the scintillator plane. At the original *L* = 1.5 m position the dispersion is approximately six times finer, of order 10 meV µm^−1^. The transverse FEL beam size on the Si(220) crystal was ∼200 µm FWHM (full width at half-maximum) under the conditions of the experiments reported here.

At the original 1.5 m detector position, the calibrated dispersion projects the ∼24 eV third-harmonic FWHM bandwidth over ∼2.3 mm on the scintillator, with the full displayed spectral range (about three FWHM) covering of order 7 mm. Combined with the typical third-harmonic pulse energy of order a few microjoules and the finite light yield and collection efficiency of the Ce:YAG/imaging chain, the signal per pixel under these conditions is too low to permit reliable single-shot measurement. This low signal per pixel regime is the limiting factor; absorption between the beryllium exit window and the detector at 7.5 keV is negligible and does not drive the signal-to-noise limitation. Moving the imaging system to 0.25 m reduces the spectral footprint to ∼380 µm FWHM at the scintillator, so that the photon density per pixel increases by roughly a factor of six while the bandwidth remains comfortably oversampled.

The intrinsic energy resolution of the imaging chain at the 0.25 m position is set by the optical resolution of the Ce:YAG imaging system rather than by the pixel pitch of the CMOS sensor. With a calibrated dispersion of 64 meV per pixel, an effective spatial sampling of 2.21 µm per pixel at the scintillator and an optical resolution of ∼8 µm FWHM, the pixel-limited contribution to the energy resolution is ∼0.06 eV (FWHM) and the optics-limited contribution is ∼0.23 eV (FWHM) at 7.5 keV. Both lie below the upper bound of ∼0.46 eV obtained from the autocorrelation analysis in Section 4.2, which places a conservative upper limit of Δ*E*/*E* ≲ 6 × 10^−5^ at the third harmonic.

The choice of an indirect detection scheme (Ce:YAG scintillator imaged onto a pco.edge 5.5 CMOS camera) rather than a direct X-ray detector such as the PSI-developed Gotthard silicon microstrip or Jungfrau pixel detectors was driven primarily by the pixel pitch. With the calibrated dispersion of 64 meV per pixel at the 0.25 m position and an effective spatial sampling of 2.21 µm per pixel at the scintillator, the 50 µm strip pitch of the Gotthard detector would correspond to an energy sampling of ∼1.4 eV per strip and the 75 µm pixel pitch of the Jungfrau detector to ∼2.2 eV per pixel. These figures are larger than the expected single-shot SASE coherence-spike width of order 0.2 eV at 7.5 keV, so neither direct detector could resolve the spectral fine structure that the indirect imaging chain captures with its ∼0.23 eV optical resolution; the higher per-photon quantum efficiency of direct X-ray detection cannot recover the spectral information once it has been integrated over a too-large pixel. A secondary consideration is the requirement for two-dimensional imaging: the transverse-mode structure of the harmonic emission, the spatially distinct low-energy tail seen during initial 2.1 keV operation (Section 4.4) and the multimode character predicted by SASE theory (Section 2.2) are accessible only with a 2D detector, which precludes the use of the one-dimensional Gotthard but not in principle the 2D Jungfrau.

## 4. Results

### 4.1. Experimental conditions

All measurements were performed at the Aramis hard and tender X-ray beamline of SwissFEL in SASE mode running at 100 Hz. For the bandwidth studies at 2.5 keV, the FEL was tuned to a fundamental photon energy of 2.5 keV producing a pulse energy of ∼800 µJ, with the spectrometer aligned to the corresponding third harmonic at 7.5 keV, corresponding to a Bragg angle of θ_B_ ≃ 25.5° for the Si(220) reflection. For the machine-tuning example at 2.1 keV, again with a fundamental pulse energy of ∼800 µJ, the spectrometer was configured to record the third harmonic at 6.3 keV for which the Si(220) Bragg angle is θ_B_ ≃ 30.8°. The third-harmonic pulse energy was not directly measured, but is estimated to be of order a few microjoules, corresponding to a third-harmonic-to-fundamental ratio of ∼10^−3^, consistent with published measurements of nonlinear harmonic generation at other XFEL facilities (Bidhendi *et al.*, 2025 ▸). In both cases, the existing SwissFEL bent-crystal spectrometer was used with an Si(220) crystal of 75 mm radius of curvature, operated in the same reflection geometry as in standard 4–13 keV operation (Rehanek *et al.*, 2017 ▸; David *et al.*, 2021 ▸).

Single-shot spectra were recorded with the insertable Ce:YAG/pco.edge CMOS imaging system described above. For the following studies, the spectrometer dispersion was calibrated by scanning the undulator gap (and hence the undulator field strength) to vary the FEL central photon energy by ±20 eV around the nominal value in 5 eV steps, and by tracking the corresponding shift of the spectral peak on the CMOS detector. The resolution analysis in Section 4.2 is based on a set of 2400 single-shot spectra acquired under stable machine conditions. The comparison with the beamline monochromator in Section 4.3 uses several thousand FEL shots per energy point to obtain the average transmission curve, which inherently includes the central-energy jitter of the SASE pulses. All data were taken during dedicated machine-study time, with the standard Aramis beamline optics and electron-beam settings as used for user operation.

### 4.2. Single-shot measurement of the third harmonic

**Figure 2:**
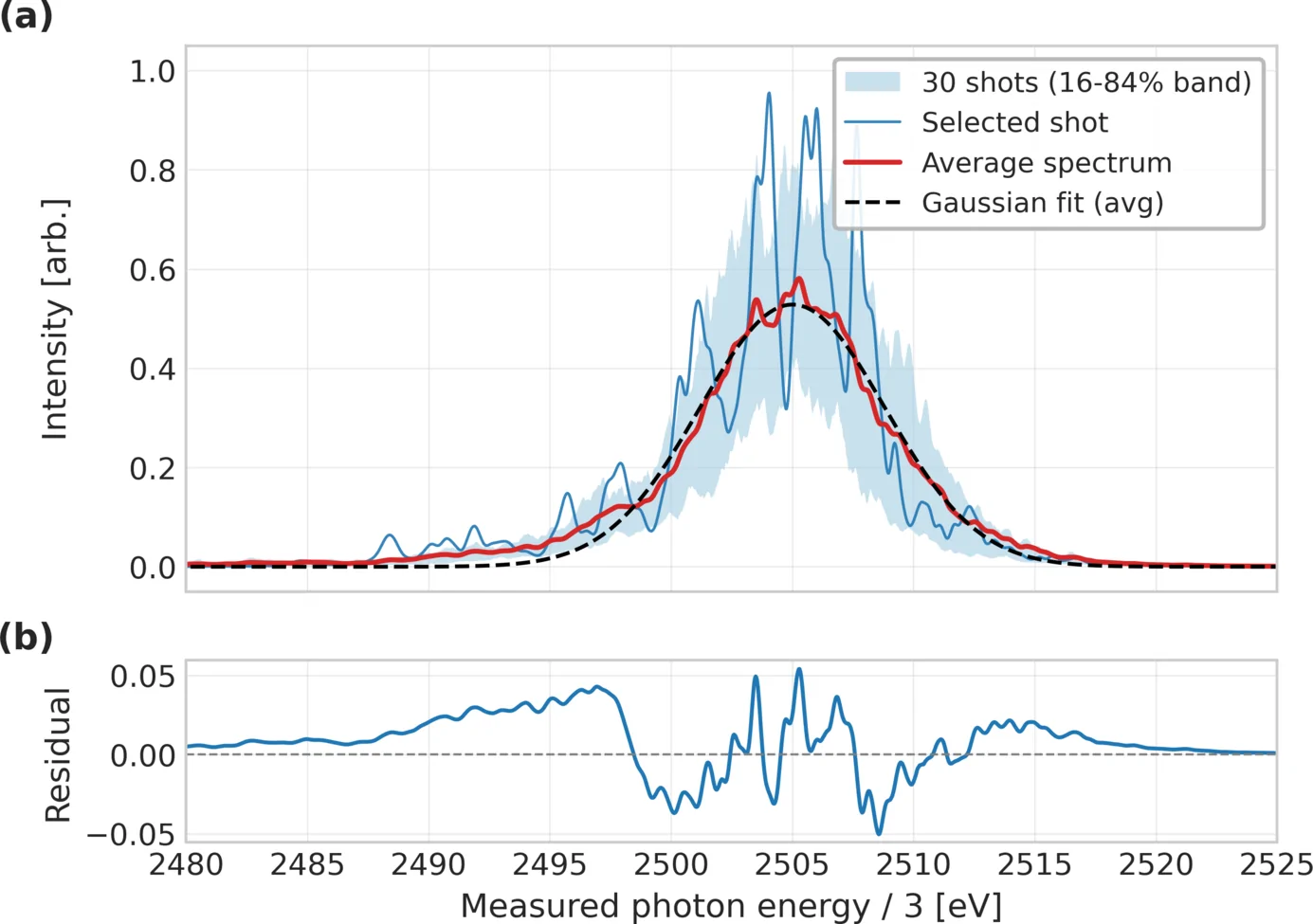
(*a*) Single-shot spectra of the third harmonic recorded with the bent-crystal spectrometer. The shaded band shows the 16%–84% range of 30 single shots and the thin blue curve shows a representative selected shot. The red curve is the average spectrum of the selected shots, and the black dashed line is a Gaussian fit to this average. The fit yields a relative energy spread of Δ*E*/*E* ≃ 3.1‰ for the fundamental at 2.5 keV. The horizontal axis is the measured third-harmonic photon energy divided by three, corresponding to the fundamental photon energy. (*b*) Residual of the average spectrum with respect to the Gaussian fit, highlighting the non-Gaussian low-energy tail.

With the spectrometer configuration and calibration as described in the previous section, we recorded single-shot spectra of the third harmonic at 7.5 keV (corresponding to a 2.5 keV fundamental). Fig. 2 ▸ shows a representative subset of these data. The shaded band indicates the 16%–84% range of 30 single shots and the thin blue curve indicates one selected shot. The red curve is the average spectrum of the selected shots, and the black dashed line is a Gaussian fit to this average.

From the Gaussian fit, we obtain an FWHM bandwidth of 7.9 eV at 2.5 keV, corresponding to a relative energy spread of Δ*E*/*E* ≃ 3.1‰. This Gaussian FWHM provides a convenient single number to characterize the bandwidth. However, the residual plot in the lower panel of Fig. 2 ▸ shows that the Gaussian systematically underestimates the intensity in the low-energy wing of the spectrum, reflecting the skewed non-Gaussian nature of the SASE pulse. Alternative measures such as the interquartile range would be less sensitive to these asymmetric tails, but for consistency with standard practice, we report the Gaussian FWHM, noting that it slightly underestimates the true spectral width.

### 4.3. Resolution

**Figure 3:**
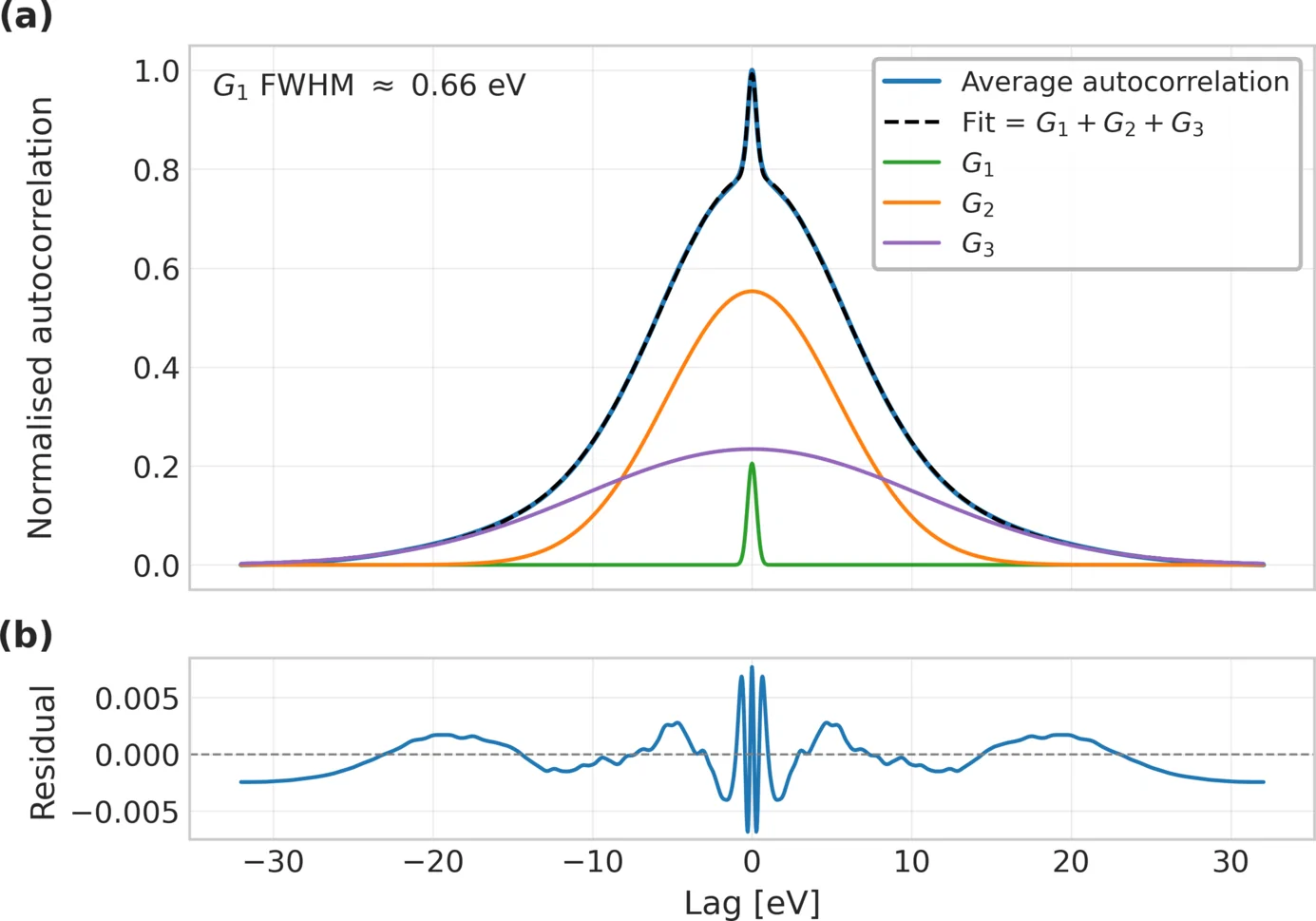
Average autocorrelation of 2400 single-shot spectra and Gaussian decomposition. (*a*) Mean autocorrelation (blue solid curve) together with the sum of three Gaussian components (black dashed curve), and the individual components *G*_1_, *G*_2_ and *G*_3_ corresponding to the average coherence spike, the single-shot bandwidth and a broad background, respectively. The narrowest component *G*_1_ is used to place an upper bound on the spectrometer resolution, while *G*_2_ provides an independent estimate of the average spectral bandwidth. (*b*) Residual of the mean autocorrelation with respect to the three-Gaussian fit.

To estimate the intrinsic resolution of the bent-crystal spectrometer, we analyse the average autocorrelation of the single-shot spectra recorded at 7.5 keV. For each of the 2400 single-shot spectra acquired under stable machine conditions (Section 3.2), we compute the normalized autocorrelation as a function of energy lag and then average these traces. The resulting mean autocorrelation is shown in Fig. 3 ▸. It exhibits a bell-shaped profile with a narrow central peak sitting on top of a broader envelope and a still wider pedestal.

The mean autocorrelation profile is well described by the sum of three Gaussian components,
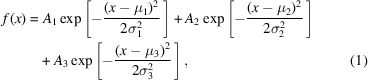
where *A*_*i*_, μ_*i*_ and σ_*i*_ are the amplitudes, centres and standard deviations of the three Gaussians (*i* = 1, 2, 3). In the fit, the components *G*_1_, *G*_2_ and *G*_3_ (ordered by increasing width) can be associated with the average single-shot coherence spike, the average single-shot spectral envelope and a broad background contribution, respectively. The fitted FWHM values are 0.65 eV for *G*_1_, 12.7 eV for *G*_2_ and 25.7 eV for *G*_3_.

For a purely Gaussian line shape, the autocorrelation is broader than the original distribution by a factor of 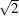 ≃ 1.4. Correcting for this, the fitted autocorrelation widths imply an average spike FWHM of ∼0.46 eV and an average single-shot spectral bandwidth of ∼9.0 eV. The latter is consistent with the FWHM of 7.9 eV obtained from the direct Gaussian fit to the average spectrum in Fig. 2 ▸, within the expected shot-to-shot fluctuations of a SASE FEL. Interpreting the 0.46 eV spike width as an upper limit on the instrumental contribution, we therefore estimate an energy resolution of Δ*E* ≃ 0.46 eV (FWHM) at 7.5 keV, corresponding to a relative resolution Δ*E*/*E* ≃ 6 × 10^−5^ at the third harmonic.

### 4.4. Monochromator comparison

**Figure 4:**
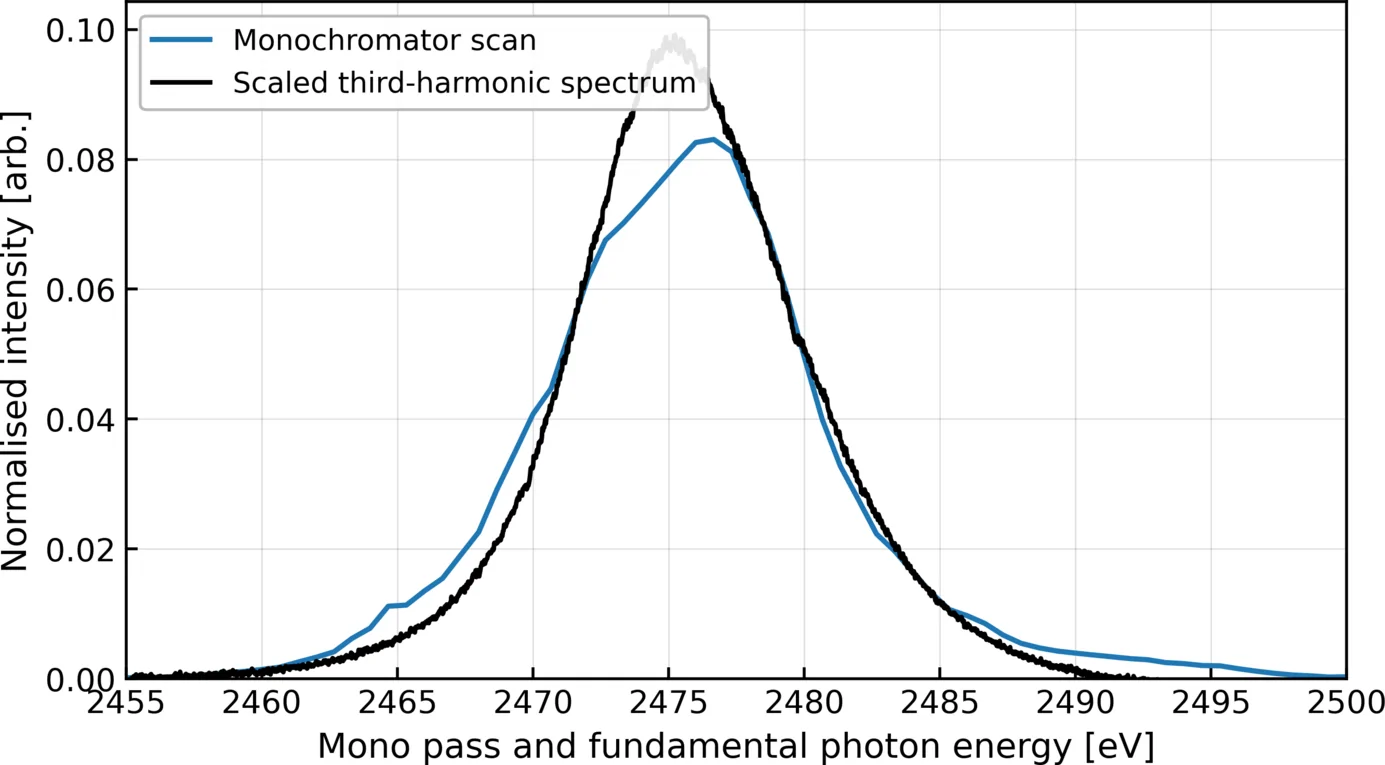
Comparison of the average spectral distribution of the 2.5 keV fundamental measured by scanning the beamline monochromator (blue curve) with the average third-harmonic spectrum measured using the bent-crystal spectrometer (black curve). For the third-harmonic spectrum, the photon-energy axis is divided by three so that both curves are plotted as a function of the fundamental photon energy. The close agreement in spectral width and shape demonstrates that the scaled third-harmonic spectrum faithfully reproduces the bandwidth of the tender X-ray fundamental.

The validity of the third-harmonic bandwidth measurement was further tested by comparing it with an independent determination using the beamline monochromator at the fundamental energy. In this measurement, the monochromator pass energy was scanned across the 2.5 keV FEL emission, and the transmitted intensity was recorded and averaged over several thousand FEL shots at each energy point. The resulting normalized transmission curve, which reflects the convolution of the FEL spectrum with the monochromator resolution and includes the intrinsic SASE central-energy jitter, is shown in Fig. 4 ▸.

The same figure also displays the average third-harmonic spectrum recorded with the bent-crystal spectrometer. For a direct comparison, the photon-energy axis of the third-harmonic spectrum is divided by three so that both traces are plotted as a function of the corresponding fundamental photon energy. The intensities of the two curves are normalized such that the integrated area under each spectrum over the displayed energy range is equal, so that the comparison highlights their spectral shape and width. Gaussian fits to the monochromator scan and to the fundamental-equivalent third-harmonic spectrum yield FWHM values of 11.12 eV and 10.98 eV, respectively. Within these experimental uncertainties and the shot-to-shot fluctuations of the SASE process, the two spectral distributions agree well in width and overall shape. This confirms that the third-harmonic spectrum, when scaled by the harmonic number, provides a reliable proxy for the bandwidth of the tender X-ray fundamental and validates the use of the bent-crystal spectrometer as an on-line diagnostic for tender X-ray operation.

### 4.5. Two-dimensional spectrometer images and transverse coherence

**Figure 5:**
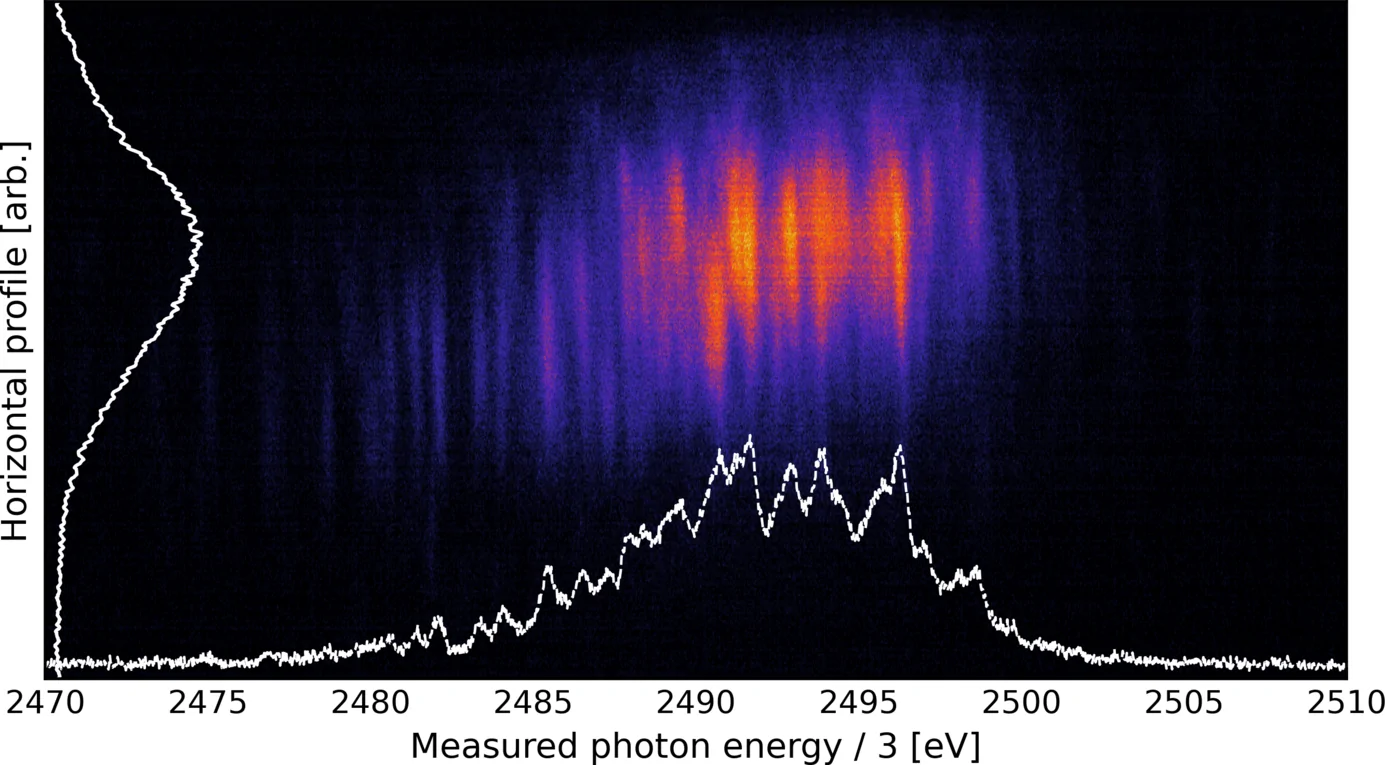
Example single-shot two-dimensional image of the third-harmonic spectrum. Photon energy is dispersed along the *x* axis, and the horizontal FEL beam profile is mapped onto the *y* axis. The image shows a non-uniform spatial structure across the full spectrum, characteristic of the multimode nature of third-harmonic SASE radiation. The low-energy part of the spectrum has a different vertical extent and mode density than the central peak, indicating emission from different sections of the electron bunch with distinct lasing conditions. The white curve shows the sum of the image over the energy axis (projection onto the transverse coordinate).

Further insight into the origin of the low-energy tail introduced in Fig. 2 ▸ is obtained by inspecting the full two-dimensional spectrometer images. Fig. 5 ▸ shows an example single-shot image of the third harmonic at 7.5 keV. The photon energy is dispersed along the horizontal (*x*) axis, while the vertical (*y*) axis corresponds to the horizontal transverse profile of the FEL beam as imaged by the spectrometer. The white vertical curve is the projection onto the vertical axis, *i.e.* the horizontal beam profile integrated over energy. The white horizontal curve is the corresponding spectral projection.

Across the full spectral range, the third-harmonic emission exhibits a non-uniform spatial distribution, which is expected for higher-harmonic SASE radiation and reflects the reduced transverse coherence and multimode character discussed in Section 2.2. A closer inspection of the image shows that individual SASE spikes are localized both spectrally and transversely, with spikes that do not extend over the entire spectral width. Instead, different parts of the spectrum are carried by different transverse modes.

Superimposed on this general graininess, the low-energy part of the spectrum around the tail shows a distinct spatial extent and mode density compared with the central peak region. The low-energy contribution occupies a different vertical region and displays a different density and distribution of bright patches, indicating that it is emitted under beam conditions that differ from those responsible for the main spectral peak. In other words, the low-energy tail is not just an extension of the central mode but is associated with a part of the electron bunch with significantly different properties.

Thus, while the one-dimensional projection in Fig. 2 ▸ already reveals a non-Gaussian low-energy tail, the two-dimensional images demonstrate that this tail is also spatially distinct and linked to a different transverse modal content. This underlines the added diagnostic value of recording full 2D spectra: they provide simultaneous access to the spectral bandwidth, the distribution of SASE spikes in energy and space, and the transverse coherence properties of the FEL pulse. Together, these offer direct visual feedback on the underlying electron-beam conditions.

### 4.6. Barometer of machine performance

Beyond providing a quantitative bandwidth and resolution, the two-dimensional spectrometer images at the third harmonic also serve as a sensitive barometer of machine performance. This was particularly useful during the setup of SwissFEL for operation at a 2.1 keV fundamental. In this configuration, the bent-crystal spectrometer was tuned to the third harmonic at 6.3 keV, and the resulting 2D spectra were monitored in real time.

**Figure 6:**
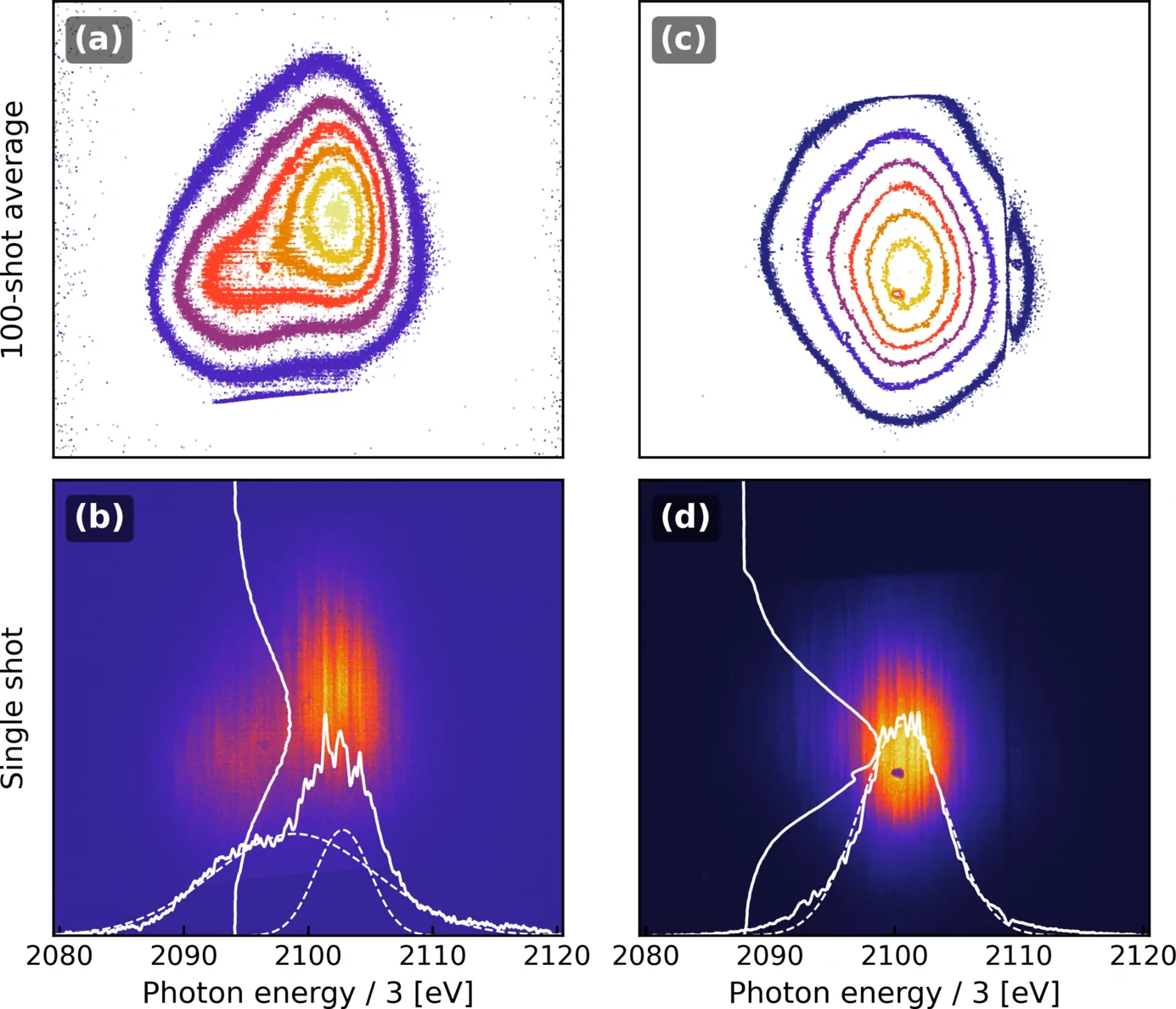
Use of the third-harmonic spectrometer as a barometer of machine performance during setup at a 2.1 keV fundamental. All panels show data recorded at the third harmonic (6.3 keV); the horizontal axis is plotted as the corresponding fundamental-equivalent photon energy (measured third-harmonic energy divided by three). (*a*) Contour plot of a 100-shot average 2D spectrum during the initial setup. The horizontal axis is the dispersed photon energy and the vertical axis is the horizontal transverse profile of the FEL beam, so that contour lines trace regions of constant shot-averaged photon density on the detector. A bimodal structure is visible: a main peak around 2105 eV and a low-energy tail between 2090 and 2100 eV, with a transverse extent and intensity distribution distinct from the main peak. (*b*) One-dimensional spectrum obtained by integrating panel (*a*) along the transverse coordinate, showing the bimodal profile but not the underlying spatial inhomogeneity. (*c*) Contour plot of an equivalent 100-shot average after optimization of the machine settings, displaying a more uniform single-mode distribution. (*d*) One-dimensional spectrum obtained by integrating panel (*c*) along the transverse coordinate, which is close to Gaussian. Together, these panels show how the 2D spectrometer images provide rapid on-line feedback on both spectral and transverse coherence properties, enabling efficient optimization of the FEL.

Fig. 6 ▸(*a*) shows a 100-shot average two-dimensional image of the third-harmonic spectrum at the beginning of the setup. As in Fig. 5 ▸, the photon energy is dispersed along the horizontal axis and the horizontal FEL beam profile is mapped along the vertical axis; contours indicate lines of constant shot-averaged photon density on the detector, so that the spatial structure of the spectrum can be read directly. The horizontal axis is plotted as the fundamental-equivalent photon energy, *i.e.* the measured third-harmonic energy divided by three. A clear bimodal structure is observed: a main peak around 2105 eV and a pronounced low-energy tail between ∼2090 and 2100 eV, with a different transverse extent and intensity distribution. The corresponding one-dimensional projection in Fig. 6 ▸(*b*) (white curve) already indicates a bimodal spectral profile, but it does not reveal the strong spatial inhomogeneity of the low-energy component. In other words, a purely 1D spectrum would have flagged a broadened or double-peaked distribution, but would have obscured the fact that different parts of the spectrum are associated with different transverse modes.

The 2D information proved crucial for machine tuning. The non-uniform multi-lobed pattern of the low-energy tail in Fig. 6 ▸(*a*) pointed to suboptimal compression of the electron beam, consistent with the reduced transverse coherence and multimode structure expected for third-harmonic SASE radiation (Section 2.2). Using the live spectrometer image as feedback, machine operators were able to adjust the compression settings and other beam parameters to minimize the spatially separated low-energy contribution.

After optimization, the contour plot in Fig. 6 ▸(*c*) shows a much more uniform single-mode distribution of the third-harmonic emission, and the corresponding 1D spectrum in Fig. 6 ▸(*d*) approaches a near-Gaussian profile. A single-Gaussian fit to the spectra indicates a reduction of the effective FWHM from 12.66 eV in the pre-optimization case [Fig. 6 ▸(*b*)] to 8.17 eV after optimization [Fig. 6 ▸(*d*)]. In the pre-optimization case this single-Gaussian fit actually underestimates the true bandwidth, since the spectrum is clearly non-Gaussian with a pronounced low-energy wing, so the improvement in spectral quality is even more significant than the FWHM values alone suggest. This illustrates how the 2D spectrometer acts as a rapid on-line diagnostic of both spectral and transverse coherence properties. Importantly, this optimization was achieved without performing any monochromator energy scans. Without such a real-time 2D diagnostic, reaching a comparable level of tuning would have required multiple slow monochromator scans over the spectral bandwidth, significantly reducing the efficiency of the machine setup, especially during short commissioning shifts. The advanced 2D spectral imaging provided by the bent-crystal spectrometer is therefore an invaluable tool for maintaining high performance and reproducible beam conditions in tender X-ray FEL operation.

## 5. Conclusions

We have demonstrated that a bent-crystal spectrometer originally developed for hard X-ray single-shot diagnostics at SwissFEL can be adapted to provide high-fidelity third-harmonic spectral measurements for tender X-ray FEL operation. By inserting an additional imaging setup at 0.25 m from the existing Si(220) crystal, we obtain sufficient dispersion and signal per pixel to record single-shot spectra of the third harmonic at 6.3 and 7.5 keV, corresponding to fundamental photon energies of 2.1 and 2.5 keV, respectively, in the tender X-ray range. The measured spectra yield a relative bandwidth of Δ*E*/*E* ≃ 3.1‰ at 2.5 keV and, from an autocorrelation analysis of 2400 shots, an upper bound on the instrumental resolution better than 6 × 10^−5^ at 7.5 keV.

The two-dimensional spectrometer images provide additional information beyond a one-dimensional spectral profile. As expected for higher-harmonic SASE radiation, the third-harmonic emission exhibits reduced transverse coherence, with non-uniform spatial structure across the full spectrum. At the same time, the low-energy wing of the spectrum shows a distinct transverse extent and mode density compared with the central peak, highlighting spectral components that arise under different beam conditions. These 2D images therefore offer simultaneous access to the spectral bandwidth, the distribution of SASE spikes in energy and space, and the transverse structure of the radiation.

We have validated the use of the third harmonic as a proxy for the tender X-ray fundamental by comparing the scaled third-harmonic spectrum with independent measurements with the beamline monochromator at 2.5 keV. The good agreement in spectral width and shape confirms that the third-harmonic bandwidth, when divided by the harmonic number, faithfully reproduces the fundamental bandwidth. In addition, the 2D spectrometer images have been shown to act as a sensitive barometer of machine performance during operation at 2.1 keV: they enabled rapid identification of a spatially separated low-energy component and guided adjustments to the compression and beam settings, leading to a single-mode near-Gaussian spectrum.

The method relies only on the natural third-harmonic emission and an existing bent-crystal spectrometer, so it does not require changes to the primary beam path or additional in-vacuum optics. In the measurements reported here the bent crystal was operated in an edge-clipping geometry during tender X-ray user operation, intercepting only the periphery of the FEL beam so that the bulk of the fundamental was delivered to the experiment while the third-harmonic spectrum was recorded in parallel. A fully non-invasive implementation, equivalent to the standard hard X-ray bent-crystal spectrometer architecture, can be obtained by adding a suitable thin beam-sampling grating upstream of the bent crystal. The approach is therefore straightforward to implement and can, in principle, be transferred to other tender X-ray configurations and to facilities that already employ similar bent-crystal spectrometer diagnostics, providing a practical on-line diagnostic tool for characterizing and optimizing tender X-ray FEL pulses.
